# Prevalence of Controlled Attenuation Parameter (CAP)-Assessed Hepatic Steatosis as a Possible Indicator of Metabolic Dysfunction-Associated Fatty Liver Disease (MAFLD) Risk in Healthy Medical Students: A Cohort Study From South India

**DOI:** 10.7759/cureus.92747

**Published:** 2025-09-19

**Authors:** Geeta S Desai, Sachi Hajare, Sandesha Ghorpade, Santosh Hajare

**Affiliations:** 1 Department of Gastroenterology and Hepatology, KLE's Dr. Prabhakar Kore Hospital and Medical Research Centre, Belagavi, IND; 2 Department of Gastroenterology and Hepatology, Jawaharlal Nehru Medical College, KLE Academy of Higher Education and Research, Belagavi, IND

**Keywords:** controlled attenuation parameter (cap), fatty liver, fibroscan, hepatic steatosis, liver fat marker, liver stiffness measurement, metabolic dysfunction associated fatty liver disease, prevalence, south india

## Abstract

Background: The prevalence of hepatic steatosis is increasing worldwide, and it is now recognized as a leading hepatic sign of metabolic syndrome. The optimal approach for screening hepatic steatosis in asymptomatic populations remains unclear. The controlled attenuation parameter (CAP), measured via transient elastography, has emerged as a non-invasive, reliable tool for quantifying liver fat. Our study aimed to determine the prevalence of CAP-assessed hepatic steatosis among healthy medical students in South India.

Methods: In this cohort study, 688 medical students aged ≥18 years with no significant alcohol consumption underwent transient elastography (FibroScan®; Echosens, Paris, France) to assess liver stiffness measurement (LSM) and CAP. Hepatic steatosis was defined as CAP ≥238 dB/m. Anthropometric parameters were recorded, and correlations with CAP were evaluated using Spearman’s coefficient. Independent predictors were identified via multivariable linear regression.

Results: The mean participant age was 20.5 ± 1.1 years; 60.6% were female. The prevalence of CAP-assessed liver steatosis, defined as CAP ≥238 dB/m, was 23.1% (n=159). CAP values showed significant correlations with body mass index (BMI) (r=0.38, p<0.001), waist circumference (r=0.37, p<0.001), and hip circumference (r=0.32, p<0.001). Multivariable analysis identified BMI as the only independent predictor of CAP (β=4.78, p<0.001).

Conclusion: The findings of this study show that hepatic steatosis is prevalent even among young, evidently healthy adults. CAP measurement using FibroScan cannot substitute for a full MAFLD diagnosis without metabolic criteria; however, it can be an effective, non-invasive tool for early detection of hepatic steatosis and may have utility in screening asymptomatic populations.

## Introduction

Hepatic steatosis is characterized by fat accumulation in more than 5% of hepatocytes in individuals without significant alcohol intake [1-3]. It is regarded as the hepatic mark of metabolic syndrome and has become increasingly prevalent alongside the global obesity epidemic. Hepatic steatosis is projected to become one of the leading causes of liver transplantation within the coming decade [4,5].

Liver biopsy remains the diagnostic gold standard for hepatic steatosis; however, its invasive nature makes it unsuitable for large-scale screening [6,7]. Conventional abdominal ultrasonography, although widely available, has limited sensitivity and can reliably detect steatosis only when hepatic fat content exceeds approximately 30% [8,9]. Advanced imaging modalities, such as computed tomography (CT), offer higher diagnostic accuracy but are costly and impractical for population-level screening [10].

The controlled attenuation parameter (CAP), derived from transient elastography (TE), has emerged as a promising non-invasive biomarker for quantifying hepatic steatosis [11-14]. CAP measures ultrasound wave attenuation within the liver, with higher values indicating greater fat content. Compared with conventional ultrasound, CAP offers superior sensitivity for detecting mild steatosis and provides reproducible, operator-independent results [15-17]. In a landmark study, a CAP threshold of 238 dB/m demonstrated 91% sensitivity and 81% specificity for detecting steatosis involving at least 10% of hepatocytes [18].

The prevalence of hepatic steatosis among apparently healthy individuals is increasingly recognized as a public health concern due to its potential long-term hepatic and cardiovascular consequences. However, its precise frequency is challenging to determine, as estimates vary depending on the screening methods and diagnostic criteria employed [1-2]. Given the limitations of existing screening modalities and the emerging role of CAP, this study aimed to assess the prevalence of hepatic steatosis among apparently healthy medical students from Karnataka, India, by TE with CAP.

## Materials and methods

This was a single-center homogenous cohort conducted in KLE's Dr. Prabhakar Kore Hospital and Medical Research Centre, Belagavi, Karnataka, from June 9, 2025, to June 14, 2025. The study was approved by the KLE Academy of Higher Education and Research (KAHER) Ethics Committee (approval number: KAHER/EC/21-22/014). All participants were informed about the study for participation through a circular and provided written informed consent.

Study population

The study involved medical students aged above 18 years, of apparently healthy status at the time of participation, from South India who voluntarily consented to undergo TE for liver stiffness measurement (LSM) and CAP assessment. The Inclusion and exclusion criteria are given in Table 1.

**Table 1:** Inclusion and exclusion criteria

Inclusion criteria	Exclusion criteria
1. Age ≥18 years	1. History of chronic liver disease (including viral hepatitis)
2. Apparently healthy status at the time of participation	2. Pregnancy
3. No significant alcohol consumption (>20 g/day)	3. Unreliable TE results, defined as fewer than 10 valid measurements from the same hepatic location or an interquartile range (IQR)/median ratio >30%.

Sample size calculation

The total number of participants required for the study was determined a priori using GraphPad StatMate 2.0 (Dotmatics, Boston, Massachusetts, United States), assuming an expected correlation of r = 0.40 between CAP and BMI, which yielded 99% power at a two-tailed significance level of α = 0.05, thereby ensuring adequate statistical power to detect clinically meaningful associations. A total of 688 participants were included in the study based on this.

Data collection

Height, weight, waist circumference, and hip circumference were measured using standardized protocols. Body mass index (BMI) was calculated as weight (kg) divided by height squared (m²). Blood pressure was recorded using an automated sphygmomanometer after five minutes of rest. No blood samples were collected, and only anthropometric and non-invasive tests were performed.

Transient elastography procedure

All eligible participants underwent TE using the FibroScan® system (FIBROSCAN 430 MINI; Device ID-03662264001192; last calibration: August 18, 2024; Echosens, Paris, France). This non-invasive method applies low-frequency (50 Hz) mechanical vibrations through an ultrasound transducer probe mounted on a vibrator. These vibrations generate a shear wave that propagates through the liver; wave velocity correlates with liver stiffness, expressed in kilopascals (kPa).

Participants were examined in the supine position with the right arm elevated to facilitate access to the intercostal spaces. Measurements were taken from the right hepatic lobe at a depth of 25-65 mm using the M-probe. Hepatic steatosis was quantified using CAP values (100-400 dB/m), calculated as the median of valid acquisitions. A CAP ≥238 dB/m indicated hepatic steatosis [11,18].

Data analysis

Data were analyzed using SPSS Statistics for Windows, version 17.0 (SPSS Inc., Chicago, Illinois, United States). Continuous variables are expressed as mean ± standard deviation (SD), and categorical variables as frequencies and percentages. Correlations were assessed using Spearman’s coefficient. Independent predictors of CAP were identified using multivariable stepwise linear regression, with all baseline anthropometric and clinical variables entered as covariates. A p-value <0.05 was considered statistically significant.

## Results

A total of 688 participants (417 females, 271 males) with a mean age of 20.5 ± 1.1 years were included. The demographic characteristics of the patients are given in Table 2.

**Table 2:** Baseline characteristics of study participants (N = 688) Values presented as mean±SD, except for sex, which is presented as n (%)

Variables	Values	Range
Age (years), mean±SD	20.5 ± 1.1	18–25
Male sex, n (%)	271 (39.4%)	—
Female sex, n (%)	417 (60.6%)	—
Height (cm), mean±SD	163.4 ± 8.2	148–186
Weight (kg), mean±SD	58.9 ± 10.7	41–95
Body mass index (kg/m²), mean±SD	22.1 ± 3.5	16.5–32.4
Waist circumference (cm), mean±SD	75.1 ± 8.2	60–102
Hip circumference (cm), mean±SD	92.1 ± 7.5	75–116
Waist-to-hip ratio, mean±SD	0.78 ± 0.05	0.70–0.98

LSM and CAP

The mean CAP value was 205.6 ± 43.8 dB/m (range: 100-292 dB/m). Using a CAP threshold of ≥238 dB/m to define hepatic steatosis, it was seen that 23.2% (n=26) of participants met the criteria for TE-defined hepatic steatosis. The mean LSM among all participants was 4.7 ± 0.9 kPa (range: 2.6-6.8 kPa), as presented in Table 3. No significant associations were observed between LSM values and the baseline characteristics.

**Table 3:** Liver stiffness measurement and controlled attenuation parameter CAP: controlled attenuation parameter; LSM: liver stiffness measurement

Parameter	Mean ± SD	Range	Threshold/Cut-off	Participants (%)
CAP (dB/m)	205.6 ± 43.8	100–292	≥238 dB/m (hepatic steatosis)	26 (23.2%)
LSM (kPa)	4.7 ± 0.9	2.6–6.8	—	No significant associations with baseline characteristics

The correlation between CAP values and anthropometric parameters is presented in Table 4.

**Table 4:** Correlation between CAP values and anthropometric parameters CAP: controlled attenuation parameter

Parameters	r (Spearman)	p-value
Body mass index (kg/m²)	0.40	<0.001
Waist circumference (cm)	0.39	<0.001
Hip circumference (cm)	0.34	<0.001
Waist-to-hip ratio	0.15	0.07

Multivariable regression analysis

In the multivariable linear regression model including all baseline variables, only BMI remained an independent predictor of CAP values (β = 4.82, 95% CI: 3.35-6.29, p < 0.001), as shown in Table 5. Waist circumference also showed a statistically significant association (p = 0.003), but its effect size was smaller than that of BMI.

**Table 5:** Multivariate linear regression analysis for predictors of CAP values β: unstandardized regression coefficient; SE: standard error; CI: confidence interval; CAP: controlled attenuation parameter

Variable	β coefficient (SE)	95% CI for β	p-value
Body mass index (kg/m²)	4.82 (0.75)	3.35 to 6.29	<0.001
Waist circumference (cm)	0.94 (0.31)	0.33 to 1.55	0.003
Hip circumference (cm)	0.68 (0.38)	-0.07 to 1.43	0.075
Waist-to-hip ratio	28.1 (17.9)	-7.0 to 63.2	0.118

## Discussion

This study shows that hepatic steatosis is present in nearly one-quarter of apparently healthy young adults, as determined by TE using the CAP. Importantly, BMI emerged as the strongest independent predictor of CAP values, even in individuals without obesity. These findings emphasize the growing burden of hepatic steatosis among young, asymptomatic populations, and support the review by Teng et al. [19], which analysed the global incidence and prevalence of nonalcoholic fatty liver disease, and the review by Wong et al., which focused on the Asia-Pacific region [20].

CAP has emerged in recent years as a widely used, non-invasive method to detect and quantify hepatic steatosis. Meta-analyses demonstrate that CAP correlates well with histological steatosis grades, especially for mild to moderate levels of steatosis, with area under the ROC curves (AUROC) often exceeding 0.80 for S ≥ 1 and S ≥ 2 levels of fat infiltration [3,5]. For example, one large meta-analysis of chronic liver disease patients reported summary sensitivities and specificities of about 0.78-0.85 for CAP in detecting ≥ S1 and ≥ S2 steatosis, respectively, though performance dropped for severe steatosis (≥ S3) [5]. However, CAP's accuracy tends to decrease as the degree of fat infiltration increases, and its performance is also influenced by patient characteristics like BMI, probe type, and etiology of liver disease [4,1]. Thus, while CAP offers a practical and relatively accurate screening tool for assessing liver fat in larger populations, its limitations in sensitivity or specificity for higher grades should inform cautious interpretation and may necessitate confirmation with more definitive diagnostics in certain cases [21,22].

The present study used CAP-based TE as an effective screening modality to assess MAFLD risk in the community. Similar to our findings, prior studies have reported that TE can detect steatosis in individuals with normal ultrasonography results [3,4]. Conventional ultrasonography is widely used for liver fat screening; however, it is operator-dependent, influenced by equipment quality, and lacks sensitivity for mild steatosis [5]. In contrast, CAP offers quantitative, reproducible measurements and is less affected by hepatic inflammation or fibrosis [6- 8].

The high prevalence of hepatic steatosis observed in this cohort aligns with global trends showing a steady rise in fatty liver disease among younger age groups [1,2]. The burden of hepatic steatosis appears particularly concerning in South Asian populations, where the epidemiological transition has been characterized by rapid urbanization, dietary shifts, and increasing rates of metabolic risk factors at relatively younger ages [19,20]. South Asian populations are predisposed to insulin resistance and central adiposity even at lower BMI thresholds compared to Western populations, which may partly explain the higher susceptibility to metabolic dysfunction-associated fatty liver disease (MAFLD). Recent regional data also highlight a growing prevalence of hepatic steatosis detected through TE and CAP, supporting its role as a feasible community-level screening tool in resource-limited healthcare settings [21,22]. These findings underscore the need for early detection strategies and culturally tailored public health interventions to mitigate the long-term hepatic and cardiovascular consequences of MAFLD in South Asia, where the disease burden is projected to rise substantially in the coming decades.

The independent association between BMI and CAP values in our study mirrors results from earlier epidemiological work [9,10], suggesting that even modest elevations in BMI may indicate increased hepatic fat content. Waist circumference was also significantly correlated with CAP in univariate analysis, underscoring the role of central adiposity in MAFLD pathogenesis. However, its predictive value diminished when adjusted for BMI, consistent with findings from other cohorts [11].

Early identification of hepatic steatosis in young adults is clinically relevant, as hepatic steatosis can progress silently to advanced fibrosis, cirrhosis, and hepatocellular carcinoma [12,13]. Moreover, hepatic steatosis is associated with an increased risk of cardiovascular disease and metabolic comorbidities [14]. Detecting the disease at an early stage provides an opportunity for lifestyle interventions, which have been shown to improve both hepatic and cardiometabolic outcomes [15-17].

Our study includes the use of a standardized, validated TE protocol and the evaluation of a homogenous, apparently healthy population. The study had sufficient power to detect a true effect of clinically meaningful correlations.

Limitations and future directions

However, several limitations should be acknowledged. First, a liver biopsy was not performed due to ethical considerations. Second, serum biochemical parameters (e.g., aminotransferases, lipid profile, fasting glucose) were unavailable for a majority of participants, precluding formal metabolic syndrome assessment. Third, ultrasonography was not used for comparison; although TE is more sensitive, cost-effectiveness analyses comparing both modalities in screening programs are warranted. Fourth, the voluntary nature of participation may have introduced selection bias. Finally, CAP measurements were obtained only once; cohort studies are needed to evaluate temporal changes in hepatic steatosis in this population. Thus, the results must be interpreted with caution. The voluntary nature of participation may have excluded higher-risk individuals and introduced selection bias, potentially underestimating the true prevalence of hepatic steatosis. Moreover, the absence of metabolic and biochemical profiling limits the ability to distinguish NAFLD from MAFLD, and therefore, any claims regarding metabolic risk remain speculative rather than definitive. The lack of a histological gold standard or comparator imaging modality further restricts diagnostic certainty.

Further research should explore the utility of CAP-based TE screening in community and primary care settings, particularly among individuals with risk factors such as elevated BMI or central adiposity. Prospective longitudinal studies could clarify whether young adults with CAP ≥238 dB/m are at greater risk for progression to metabolic dysfunction-associated steatohepatitis (MASH) or advanced fibrosis. Additionally, research should determine optimal CAP cutoffs for different ethnicities, considering genetic, dietary, and lifestyle variations.

## Conclusions

The findings of this study show that hepatic steatosis is prevalent among young, apparently healthy adults, with nearly one-quarter of this cohort showing TE-defined hepatic steatosis. BMI emerged as the most significant independent predictor of CAP values, even in non-obese individuals. These findings underscore the growing recognition of fatty liver disease as an emerging public health concern, even in populations traditionally considered at low risk. However, it should be noted that the participants were medical students from a single center in South India, and the cohort was relatively homogeneous (young, educated, health-conscious). Also, while CAP offers a practical, noninvasive tool for early detection and may play an important role in screening strategies, particularly where access to advanced diagnostics is limited, CAP-detected steatosis cannot, currently, substitute for metabolic profiling in indicating MAFLD risk.
